# The area-based social patterning of injuries among 10 to 19 year olds Changes over time in the Stockholm County

**DOI:** 10.1186/1471-2458-8-131

**Published:** 2008-04-23

**Authors:** Anne-Mari Reimers, Antonio Ponce de Leon, Lucie Laflamme

**Affiliations:** 1 Karolinska Institutet, Department of Public Health Sciences, Division of Social Medicine, SE-171 76 Stockholm, Sweden; 2 Centre for Public Health, Norrbacka, SE-171 76 Stockholm, Sweden; 3 Karolinska Institutet, Department of Public Health Sciences, Division of International Health, SE-171 77 Stockholm, Sweden; 4 Department of Epidemiology, Institute of Social Medicine, University of Rio de Janeiro State (IMS/UERJ)

## Abstract

**Background:**

Area-based studies of childhood injuries strongly suggest that neighborhood socio-demographic and economic circumstances impact on various – though not all – types of injuries. The primary aim of this study was to investigate the stability over time of the association between area characteristics and childhood injuries of various causes.

**Methods:**

Register-based and ecological, the study encompassed Stockholm County's 138 parishes, and considered two time periods (1993–95; 2003–05). Two indices were measured: economic deprivation and social fragmentation, and parishes were allocated to their respective quintile on each index. Data on both unintentional and intentional injuries for children (boys and girls) aged 10–14 and 15–19 respectively were gathered from the County Council's hospital inpatient register. For each period and index, gender, age and cause-specific comparisons were made to assess the rate ratios (with 95% confidence intervals) of being injured using parishes belonging to the best index level as a comparison group. A series of simple and partial Pearson correlations were also calculated to assess the independent contribution of each index.

**Results:**

Regardless of time period, there were rather few significant rate ratios and, when they occurred, there were both under and excess risks. For instance, in each period, boys from both age groups living in parishes with the highest levels of economic deprivation had lower rate of injury as a motor vehicle rider. Most strikingly, intentional injuries were more frequent during the second time period and in considerable excess among girls aged 15–19 from more economically deprived areas. Also, during that last period, none of the injury causes correlated significantly with the index of social fragmentation after adjustment for economic deprivation (partial correlation).

**Conclusion:**

Over a ten-year period, differential economic deprivation among parishes has widened more than social fragmentation in Stockholm County. The correlation between those indices is high in both periods of time whilst the association between the levels of each index and injury rates varies depending on group of injuries or time period considered. It is of concern that intentional injuries have increased numerically and are significantly and positively correlated with economic deprivation (net of social fragmentation), in particular among girls.

## Background

Injuries, both intentional and unintentional, are responsible for a great deal of child and adolescent mortality in all parts of the world [1,2]. In Europe in particular, data comparing the mid-1980s and the mid-1990s show that the burden of injury mortality has worsened in some countries, in spite of clear downward trends [3,4]. In addition, injuries are unequally distributed across European countries, with high income ones registering far less casualties than others [5,6].

At country level, there is ample evidence showing that, for injury causes like traffic, inter-personal violence and self-inflictions, there may be substantial differences between groups of people and even between geographical areas [7-10]. In Sweden, where the current study was conducted, the downward trend in injury mortality is very pronounced [3,4]. In contrast with many other – and neighboring – countries, socioeconomic differences are not so marked in early childhood but they get quite pronounced with age, in particular in the second decade of life [11-13]. Also, socioeconomic differences exist among both male and female children [11,12,14,15].

Studies on places and health demonstrate that living conditions represent an important avenue of explanation for the social patterning of children and adolescent health and safety. Indeed deprivation and low socioeconomic status in the living area have been found to be associated for example with higher rates of diabetes [16] and obesity [17]. In the UK, it is also associated with suicide and parasuicide outcomes [18-20] although in one study no association between deprivation and suicide was found either before or after adjusting for social fragmentation and psychiatric admission rates [21]. When it comes to unintentional injuries, area-based studies conducted in the UK [22-26], New Zealand [27], Canada [28,29], USA [30-32], Brazil [33] and Sweden [34,35] strongly suggest that neighborhood socio-demographic and economic circumstances impact on various – though not all – types of injuries. This is not surprising as the likelihood of injuries happening is not only determined by individual characteristics but also by environmental ones (e.g., amount of hazards, availability and affordability of post-trauma care preparedness). In the specific case of road traffic injuries, besides deprivation [36] even parameters like busier streets and/or greater traffic volume are associated with increased injury risks [37]. And low- and middle-class neighbourhoods have been found to register more injuries not only on pavements and in streets but also in playgrounds.

The identification of area characteristics associated with higher risk levels offers relevant targets for prevention [38,39]. One obvious example is the association between characteristics of the road traffic environment at the area level and childhood road traffic injuries and where changes in the physical environment (e.g., traffic separation and traffic calming measures) can improve the safety level of poorer areas [36,40]. Besides that, addressing social features of the neighborhood other than its physical characteristics may also contribute to risk reduction, e.g., walking school buses although sometimes the uptake of such measures is easier in affluent neighbourhoods than in deprived ones [41].

Besides area differences, one important dimension to pay attention to when determining a safety promotion agenda is whether the social patterning across areas is stable over time or, rather, subject to change. Surprisingly few studies have been published that address changes in socioeconomic differences in injuries over time [2-4,42]. Most of them – referred to above – are from the UK and point to increasing gradients. In Sweden, changes over time have not been studied much-except for some health studies in adults [43,44]. This aspect does deserve social fragmentation special consideration. Indeed, in the early and mid-1990s, Swedish society went through great changes after a boom in the economy during the second part of the 1980s. A deep recession in the early 1990s lead to unemployment, living costs for families with children increased and so on. So far, the 2000s have been characterised by steady economic growth. But improved economic conditions and a positive development in labour market have not resulted in improved health in the adult population [45].

Against this background, the primary aim of this study is to shed light on the stability over time of the association between area characteristics and childhood injuries of various causes. This is going to be achieved by first examining parish-based differences in socioeconomic deprivation and social fragmentation for two distinct time periods and then considering whether (profile of differences) changes over time are observed. The analysis will focus on the second decade of life – where both injury rates and socioeconomic differences are on the increase in Sweden – and children will be split by sex and two age groups and attention will be paid to five causes of unintentional and intentional injuries. Thirdly, a series of simple and partial Pearsson correlations are measured to assess the independent contribution of socioeconomic deprivation and social fragmentation scores.

## Methods

### Area characteristics

The study was register-based and ecological. Two time periods with an interval of 10 years (1993–95 and 2003–05) were used. Periods of time were chosen rather than years as a continuous variable since the number of injuries by years was relatively low and the change of ICD codes in 1997 made the code series very unreliable for a number of years. The analyses included the whole Stockholm County region and comparisons were made at the parish level. Usually, parishes jointly make up a Swedish municipality, but since they are also the fundamental administrative and working units within the Church of Sweden, there may not be perfect correspondence. In 1994, Stockholm County had a population of 1.71 million and 10.5% were in the age range 10–19 years; in 2004, the population had risen to 1.87 million and 12.4% were aged 10–19 years. Parish characteristics were extracted from the Office of Regional Planning and Urban Transportation and Statistics Sweden's data sets for the years 1994 and 2004 respectively and the parish divisions of 1999 (138 parishes) were used for the two periods.

Socioeconomic deprivation was measured using three of the four variables included in the Townsend index developed in the United Kingdom [46,47]: (*i*) proportion of dwellings that are not owner-occupied; (*ii*) proportion of households with no car; (*iii*) proportion of unemployed in age group 18–64 years. Overcrowding was not considered as it is not a significant problem in Sweden in general and in Stockholm in particular [48]. For each study period, the z scores for each parish were calculated using the mean and standard deviation of each variable (see Table 1). The z scores of the variables were summed by parish, higher positive scores indicating greater socioeconomic deprivation. In 1994, the economic deprivation scores ranged from -6.3 to 4.7 and in 2004, from -6.9 to 4.9.

**Table 1:** Descriptions of the variables included in the compilation of the two indices. Reference periods 1994 and 2004. All expressed in percentages (%).

		**1994**				**2004**			
Variable	Description	Mean	Std	Min	Max	Mean	Std	Min	Max
**Townsend index**									
Unemployment	Percentage of economically active residents aged 18–64 who are unemployed.	5.4	1.7	1.1	9.7	2.8	1.0	0.0	6.9
Car ownership	Percentage of private households who do not posses a car.	48.4	16.4	16.8	89.8	45.5	16.0	27.1	78.8
Home ownership	Percentage of private dwellings not owner occupied.	48.4	28.3	0.0	99.6	29.2	25.8	0.0	80.9
Overcrowding	Percentage of private households with children with >2 people per room, kitchen and 1 room uncounted.	--	--	--	--	--	--	--	--
**Index values**		**0.0**	**2.5**	**-6.3**	**4.7**	**0.0**	**2.6**	**-6.9**	**4.9**
**Congdon index**									
Private renting	Percentage of private households in rented accommodation.	48.4	28.3	0.0	99.6	29.2	25.8	0.0	80.9
Single person household	Percentage of residents aged <65 and living alone.	45.5	13.6	25.2	81.9	48.6	12.2	28.8	81.0
Unmarried	Percentage of residents aged >15 and not married.	46.9	8.7	22.3	61.4	43.1	7.9	22.2	58.5
Mobility	The percentage of residents that moved in or out during the previous year.	23.1	7.3	10.4	39.7	23.9	6.7	8.0	40.9
**Index values**		**0.0**	**1.9**	**-4.0**	**4.0**	**0.0**	**2.0**	**-4.4**	**4.5**

Social fragmentation was derived from the four variables used to compile the Congdon index [49]: (i) mobility; (ii) proportion of single-person households; (iii) proportion of non-married adults; (iv) proportion of persons in privately rented accommodation. Scores for each individual parish were calculated in the same manner as for economic deprivation: higher positive scores reflecting greater social fragmentation (see Table 1). The scores ranged from -4.0 to 4.0 in 1994 and from -4.4 to 4.5 in 2004, the scores span having then increased.

Table 1 summarizes each variable and each index for the two reference years (mean, standard deviation, minimum and maximum). For the index of economic deprivation, both percentage of unemployment and of home renting were substantially lower on average in 2004 than they were in 1994. Reduced unemployment could be expected as there had been a considerable recession in the first half of the 1990s; the higher percentage of home ownership was also to be expected as the decade had been marked by a strong tendency to convert rented apartments into condominiums. In that sense, the change is more a reflection of housing policy trend than improvement in wellbeing. This also explains the drop in private renting observed in the social fragmentation index. Both indices have a wider range in 2004 than in 1994.

**Figure 1:**
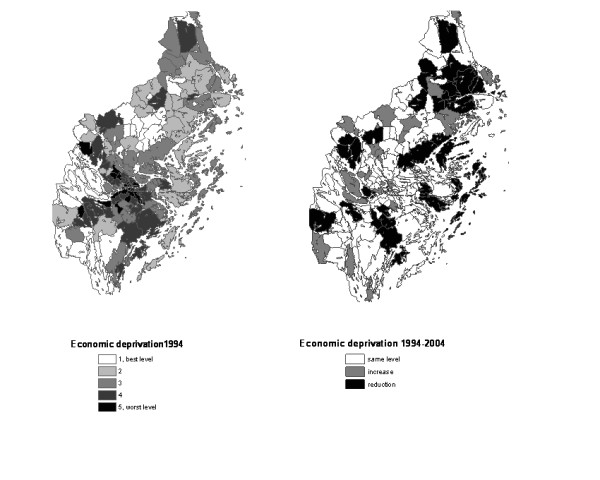
**Material deprivation at parish level.** Quintiles in 1994 and changes between 1994 and 2004.

For further analysis, each parish in both periods, 1994 and 2004, was ranked and then placed in ascending order by index and then divided into quintiles, based on this ranking. Every group had about 28 parishes and Group I constituted the least deprived – or less fragmented – group (and used as the reference group in the subsequent analyses; see below). For each index, Table 2 indicates the number of parishes that remained in the same quintile in both periods (diagonals in the tables) as well as those whose position improved or worsened. Those parishes are also illustrated in the maps presented in Figures 1a to 1d where Figures 1a and 1c show the index-specific distribution of the parishes by quintiles in 1994 and Figures 1b and 1d show the direction of the change that occurred from 1994 to 2004 (same level, higher level in 2004 and lower level in 2004).

**Table 2:** Parishes distributed to five economic deprivation and social fragmentation levels according to the reference year (numbers are presented).

		**Economic deprivation 2004**					
	Index group	1	2	3	4	5	No. of parishes
	
**Economic deprivation 1994**	1	16	9	3	0	0	28
	2	10	11	6	1	0	28
	3	1	11	13	2	1	28
	4	0	0	6	19	3	28
	5	0	0	0	5	21	26
	
	Total	27	31	28	27	25	138
		**Social fragmentation 2004**					
	Index group	1	2	3	4	5	No. of parishes
	
**Social fragmentation 1994**	1	13	10	4	0	0	27
	2	11	11	7	0	0	29
	3	3	6	14	4	1	28
	4	0	1	6	18	3	28
	5	0	0	0	5	21	26
	
	Total	27	28	31	27	25	138

**Figure 2:**
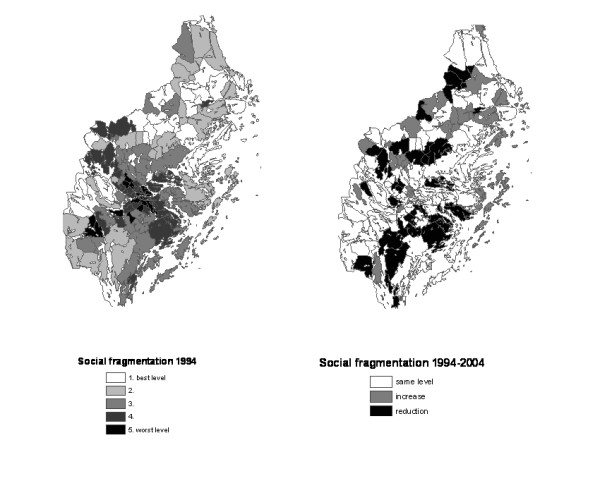
**Social fragmentation at parish level.** Quintiles in 1994 and changes between 1994 and 2004. 3a) 10–14 years. 3b) 15–19 years.

For the deprivation index, 80 of 138 parishes (58%) remained in the same quintile in both periods and for the social fragmentation index, 77 parishes (56%). A negative change from 1994 to 2004 is observed in 36 (26.0%) and 37 (26.8%) parishes respectively and a positive one is observed in 33 (23.9%) and 32 (23.2%) parishes. There is no clear geographic distribution of those parishes where changes have occurred but there is a tendency for the least deprived parishes to be located outside the center of the county and for improvements over time (lower deprivation level) to be more frequent in the northern part (see Fig. 1a and 1b). The most deprived parishes in Figure 1a are almost the same parishes as those with high social fragmentation (see Figure 2a). Figure 2b shows that the parishes with the highest social fragmentation in 2004 remain quite the same as in 1994 and that several of them are in the northern part of the county.

### Injury causes

The injuries considered were those involving children aged 10–19 years, residing in Stockholm County at the time of the injury, who were hospitalized at least one night following the injury during the two three-year periods: January 1993 – December 1995 and January 2003 – December 2005. Cases were identified from the County Council's inpatient register maintained on behalf of the Health Care Board of Stockholm County Council. The register has comprehensive coverage, including all patients who stay at least one night in any of the hospitals in Stockholm County. Missing data in Sweden's Hospital Discharge Register are very low, estimated to range between 1 and 2 percent [39]. Also, validity studies show that causes of injuries may be wrong in about 10 percent of cases. Although the possibility of differences between parishes with regard to the likelihood of keeping an injured child in hospital following an injury [40] might be of importance, there is no evidence of such bias in a Swedish setting [41].

Five causes of injuries with documented area-based differences in Stockholm County were considered, of which three were unintentional (fall, vulnerable road user and motor-vehicle rider) and two intentional (self-inflicted and violence-related). Injuries that occurred during the period 1993–95 were grouped according to the International Statistical Classification of Diseases and Related Health Problems, Version 9 and for the period 2003–05, Version 10 was used. The codes of both periods are presented in Table 3. In all instances, injuries were grouped into categories on the basis of cause of injury of main condition at first discharge, implying that any single subject could only be registered once for each cause during any period (800 children had more than one registered injury in 1993–95 and 945 in 2003–05). In total, 4 331 injuries (occasions of care) were reported in 1993–95 and 4 805 in 2003–05. There remain 4 147 and 4 463 injuries for each study period respectively after exclusion of those injuries of patients from outside Greater Stockholm, foreign patients, and those with no home address.

**Table 3:** ICD codes used in the study

**Cause of injury**	**ICD-10**	**ICD-9**
**Fall injuries**	W00–W19	E880–E888
**Vulnerable road user-related injuries**	V01–V19, V80	E819F-G, E826, E838A,D,E,X
- Pedestrian	V01–V09	E838A,D,E,X
- Cyclist	V10–V19	E819G, E826
- Animal-rider or occupant	V80	E819F
**Motor-vehicle rider-related injuries**	V20–V79, V81–V99	E819A-E,J-X, E841, E849
- Moped rider	V20–V29	E819C-E,J-X
- Car passenger	V40–V79	E819A-B
- Other land transport accident	V81–V89	
- Water-, air- or other unspec transport accidents	V90–V99	E841, E849
**Violence-related injuries**	W50–W529, X60–Y09	E950–E969
- Deliberate self-harm	X60–X84	E950–E959
- Inter-personal violence	W50–W529, X85–Y09	E960–E969

**Figure 3:**
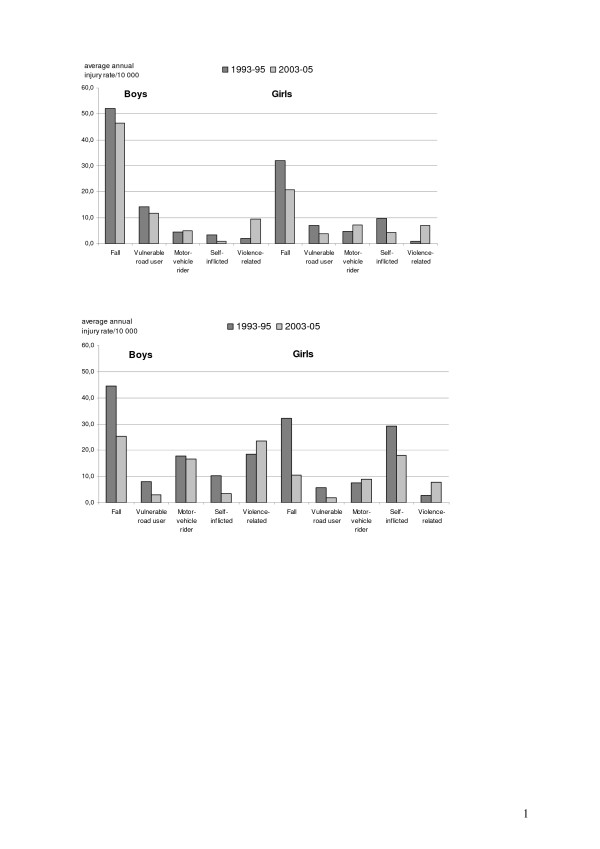
Average annual injury rates per 10 000 subjects by period and gender.

Injured subjects were then split by gender and the two age groups 10–14 (2 205 and 2 260 injuries) and 15–19 years (1 768 and 2 379 injuries). Figure 3 shows the gender and age distribution of the injury rates by cause and study period. In all instances, fall is the most frequent cause of injury, with a reduction, sometimes substantial, from the first to the second study period. Reductions over time are also observed for several causes but not for violence-related injuries among both age groups of boys and girls, and injuries as motor vehicle riders among young boys and older girls, though much less remarkably. In the age group 15–19 years, the relatively high frequency of violence-related injuries among boys and that of self-inflicted injuries among girls are also noteworthy.

### Data analysis

Using Poisson regressions models, a series of analyses specific for each index and time period were conducted by gender and age group to compare parishes from each index level with those from the best level, compiling rate ratios with 95% confidence intervals. The mid-year populations of each parish were used as the denominator terms. When the number of injuries did not exceed 60 for a given group, no regression was compiled. Boys and girls were analysed separately as there are expected differences in their respective injury patterns and life styles.

As the Pearson correlation between the two indices was high, namely in period 1993–95 it was 0.83 (p < 0.0001), and in 2003–05 it was 0.86 (p < 0.0001), further analyses were conducted. Stratifying by sex, age group and period so as to assess the simple and partial Pearson correlations between socioeconomic deprivation or social fragmentation scores and rates of every injury mechanism. Partial correlation coefficients provide the correlation between the level of economic deprivation (X) and a given injury mechanism (Y) correcting for the level of social fragmentation (Z) and vice versa (X ↔ Z). Data processing was performed using SAS (Version 9.1).

The study was approved by the Regional Ethical Review Board in Stockholm.

## Results

### Association between two area-based indices and injury rates by time period (1993–95 and 2003–05) and across time periods

Table 4 presents the gender- and age-specific rate ratios with 95% confidence intervals for various causes of injuries, comparing children from parishes from various levels of material deprivation with children from the least deprived parishes. Both in the earlier and in the more recent period, there are rather few instances where the rate ratios of children from the former parishes are significant different from those from the latter ones. When significant differences occur, both "protective" and "aggravating" associations are found, and, globally, there is no clear pattern over injury causes.

**Table 4:** Results of applying Poisson regression to various injuries by gender, age group, time period and economic deprivation. (Groups with number of injuries < 60 were not calculated)

**Economic deprivation**	**Boys 10–14 yr 1993–95**	**2003–05**	**Boys 15–19 yr 1993–95**	**2003–05**	**Girls 10–14 yr 1993–95**	**2003–05**	**Girls 15–19 yr 1993–95**	**2003–05**
Fall injuries								
**I**	1.00	1.00	1.00	1.00	1.00	1.00	1.00	1.00
**II**	1.18 (0.68–2.02)	1.32 (0.85–2.06)	0.93 (0.51–1.70)	0.80 (0.45–1.41)	0.83 (0.39–1.78)	0.96 (0.52–1.78)	0.82 (0.38–1.76)	2.36 (1.05–5.31)
**III**	1.45 (0.91–2.33)	1.17 (0.76–1.80)	1.69 (1.03–2.77)	0.81 (0.48–1.37)	1.75 (0.95–3.24)	1.02 (0.58–1.82)	0.65 (0.42–0.99)	2.87 (1.37–6.01)
**IV**	1.47 (0.92–2.34)	1.18 (0.77–1.81)	1.38 (0.84–2.27)	0.90 (0.53–1.50)	1.60 (0.87–2.95)	0.83 (0.46–1.48)	0.62 (0.41–0.94)	2.21 (1.05–4.63)
**V**	1.62 (1.01–2.58)	1.12 (0.73–1.71)	1.50 (0.91–2.48)	0.94 (0.56–1.57)	1.48 (0.80–2.75)	1.07 (0.60–1.89)	0.65 (0.42–0.99)	2.62 (1.25–5.51)
*No of injuries*	*722*	*877*	*613*	*481*	*418*	*392*	*424*	*198*
Vulnerable road user								
**I**	1.00	1.00	1.00		1.00	1.00	1.00	
**II**	1.07 (0.46–2.49)	0.65 (0.32–1.31)	1.92 (0.40–9.22)		0.72 (0.16–3.21)	0.80 (0.26–2.48)	3.94 (0.48–31.99)	
**III**	1.01 (0.48–2.12)	0.73 (0.39–1.38)	2.49 (0.60–10.34)		1.96 (0.60–6.33)	0.58 (0.20–1.71)	3.99 (0.54–29.34)	
**IV**	0.86 (0.41–1.79)	0.57 (0.30–1.08)	2.73 (0.66–11.24)		1.19 (0.36–3.90)	0.56 (0.19–1.64)	2.99 (0.40–22.139)	
**V**	0.97 (0.46–2.03)	0.53 (0.28–1.01)	1.65 (0.39–7.04)		0.70 (0.20–2.40)	0.52 (0.18–1.53)	2.19 (0.29–16.57)	
*No of injuries*	*192*	*233*	*111*	*58*	*90*	*74*	*74*	*35*
Motor-vehicle rider								
**I**	1.00	1.00	1.00	1.00	1.00	1.00	1.00	1.00
**II**	0.68 (0.26–1.84)	0.81 (0.32–2.02)	0.84 (0.47–1.49)	0.71 (0.42–1.18)	1.51 (0.54–4.19)	1.24 (0.52–3.01)	1.87 (0.52–6.81)	1.59 (0.81–3.12)
**III**	0.55 (0.24–1.26)	0.46 (0.19–1.13)	0.67 (0.41–1.10)	0.50 (0.31–0.82)	0.42 (0.15–1.17)	1.00 (0.43–2.33)	1.77 (0.55–5.75)	1.68 (0.92–3.07
**IV**	0.20 (0.08–0.51)	0.54 (0.22–1.29)	0.41 (0.25–0.69)	0.46 (0.28–0.74)	0.42 (0.15–1.13)	0.49 (0.20–1.18)	1.41 (0.43–4.60)	1.04 (0.56–1.93)
**V**	0.16 (0.06–0.43)	0.26 (0.10–0.67)	0.33 (0.19–0.57)	0.30 (0.18–0.50)	0.33 (0.12–0.95)	0.47 (0.20–1.15)	0.73 (0.21–2.52)	0.99 (0.52–1.87)
*No of injuries*	*63*	*94*	*249*	*316*	*62*	*135*	*98*	*168*
Self-inflicted								
**I**			1.00		1.00		1.00	1.00
**II**			1.19 (0.45–3.12)		2.69 (0.59–12.29)	1.00	0.82 (0.38–1.76)	4.87 (2.05–11.61)
**III**			1.15 (0.49–2.68)		3.31 (0.80–13.65)	1.05 (0.50–2.21)	1.10 (0.59–2.07)	4.41 (1.92–10.17)
**IV**			0.73 (0.31–1.73)		2.40 (0.58–9.93)	0.97 (0.46–2.03)	1.64 (0.89–3.03)	4.33 (1.89–9.93)
**V**			0.98 (0.41–2.31)		2.16 (0.52–9.02)	1.07 (0.51–2.21)	1.71 (0.92–3.17)	9.97 (4.41–22.53)
*No of injuries*	*48*	*16*	*142*	*59*	*127*	*82*	*384*	*343*
Violence-related								
**I**		1.00	1.00	1.00		1.00		1.00
**II**		3.57 (0.85–15.01)	0.99 (0.48–2.08)	1.06 (0.57–2.00)		4.53 (0.60–34.06)		2.44 (0.96–6.18)
**III**		2.53 (0.61–10.46)	0.69 (0.36–1.32)	1.10 (0.61–2.00)		5.13 (0.71–37.29)		2.66 (1.13–6.27)
**IV**		3.00 (0.73–12.31)	1.00 (0.54–1.87)	1.19 (0.66–2.15)		3.59 (0.49–26.26)		2.25 (0.69–5.29)
**V**		2.64 (0.64–10.84)	1.05 (0.56–1.98)	1.00 (0.55–1.80)		4.27 (0.59–31.06)		2.72 (1.16–6.39)
*No of injuries*	*28*	*181*	*253*	*446*	*12*	*131*	*35*	*148*

Mixed results between periods are observed for instance in the case of fall-related injuries among girls age 15–19 years: whereas girls from parishes from groups III, IV and V register significantly lower rate ratios of fall compared to girls from parishes in level I (a kind of "protective effect"), girls from parishes from all levels of deprivation register significantly higher rate ratios in the second period ("aggravating effect"). For traffic-related injuries, significant differences are observed exclusively for children injured as motor-vehicle riders where deprivation acts rather as a protective factor. Indeed, in the first period, significantly lower rate ratios are found for boys in both age groups from the parishes from group IV and V. Also, for the second period, boys 10–14 years from parishes from group V and those 15–19 years from groups III-V have significantly lower rate ratios.

Turning to intentional injuries, some aggravating effects of deprivation are observed when significant differences appear in the second reference period, among girls from the older age group only and for both diagnoses: self-inflicted injuries and those due to inter-personal violence. In the case of self-inflicted injuries, girls from parishes belonging to groups II to V have significantly greater rates than those from better-off parishes; the excess risks are very high, varying from 4.33 (group IV) to 9.97 (group V). Girls from the parishes in group III are also at excess risk of injuries due to inter-personal violence (RR = 2.66; 95% CI 1.13–6.27) and so are those from group V (RR = 2.72; 95% CI 1.16–6.39).

Table 5 presents the gender- and age-specific rate ratios with 95% confidence intervals for various causes of injuries, comparing parishes from various levels of social fragmentation with those with the best level. The patterns observed are similar in many ways to those observed for material deprivation. This is definitely the case for the two categories: violence-related injuries (aggravating effect) and for traffic-related injuries as motor-vehicle riders (protective effect), among boys. For girls in the case of motor-vehicle rider injuries, a kind of threshold is observed again in the second period where a significantly lower rate ratio is registered among young girls from parishes from groups IV and V, and those from group V, among the older ones.

**Table 5:** Results of applying Poisson regression to various injuries by gender, age group, time period and Congdon's social fragmentation

**Social fragmentation**	**Boys 10–14 yr 1993–95**	**2003–05**	**Boys 15–19 yr 1993–95**	**2003–05**	**Girls 10–14 yr 1993–95**	**2003–05**	**Girls 15–19 yr 1993–95**	**2003–05**
Fall injuries								
**I**	1.00	1.00	1.00	1.00	1.00	1.00	1.00	1.00
**II**	0.91 (0.52–1.62)	1.03 (0.68–1.54)	1.69 (0.81–3.53)	1.01 (0.59–1.73)	1.11 (0.46–2.68)	1.05 (0.61–1.81)	1.05 (0.57–1.91)	1.72 (0.78–3.80)
**III**	1.09 (0.67–1.77)	1.14 (0.78–1.67)	1.94 (0.99–3.79)	0.91 (0.55–1.51)	1.74 (0.81–3.74)	0.80 (0.48–1.33)	0.84 (0.49–1.43)	2.46 (1.23–4.94)
**IV**	1.17 (0.73–1.89)	0.96 (0.66–1.40)	2.21 (1.14–4.30)	1.05 (0.64–1.72)	2.11 (0.99–4.49)	0.83 (0.50–1.43)	0.82 (0.48–1.40)	2.23 (1.10–4.51)
**V**	1.26 (0.78–2.03)	1.00 (0.68–1.47)	2.06 (1.05–4.03)	0.95 (0.57–1.59)	1.66 (0.77–3.57)	0.99 (0.59–1.67)	0.76 (0.44–1.30)	2.22 (1.09–4.53)
*No of injuries*	*722*	*877*	*613*	*481*	*418*	*392*	*424*	*198*
Vulnerable road user								
**I**	1.00	1.00	1.00		1.00	1.00	1.00	
**II**	2.31 (0.27–19.74)	1.61 (0.72–3.60)	4.86 (1.14–20.68)		4.13 (0.52–32.57)	0.75 (0.26–2.13)	1.34 (0.14–12.93)	
**III**	3.45 (0.47–25.21)	1.24 (0.57–2.69)	2.56 (0.62–10.55)		3.80 (0.52–27.77)	0.57 (0.21–1.51)	2.58 (0.35–19.06)	
**IV**	4.24 (0.59–30.65)	1.00 (0.46–2.17)	2.77 (0.68–11.31)		2.71 (0.37–19.85)	0.41 (0.15–1.10)	2.54 (0.35–18.59)	
**V**	2.41 (0.32–17.88)	0.89 (0.40–2.01)	2.88 (0.70–11.82)		1.50 (0.20–11.37)	0.66 (0.24–1.79)	1.61 (0.21–12.20)	
*No of injuries*	*192*	*233*	*111*	*58*	*90*	*74*	*74*	*35*
Motor-vehicle rider								
**I**	1.00	1.00	1.00	1.00	1.00	1.00	1.00	1.00
**II**	2.21 (0.48–10.09)	0.85 (0.36–1.99)	0.86 (0.47–1.59)	0.74 (0.46–1.21)	2.98 (0.67–13.21)	0.94 (0.46–1.92)	3.59 (0.45–28.67)	1.01 (0.53–1.91)
**III**	1.39 (0.33–5.85)	0.48 (0.21–1.10)	0.60 (0.35–1.02)	0.55 (0.35–0.86)	1.12 (0.26–4.78)	0.62 (0.31–1.24)	3.87 (0.53–28.21)	1.22 (0.72–2.09)
**IV**	0.59 (0.14–2.60)	0.48 (0.21–1.08)	0.35 (0.20–0.60)	0.39 (0.24–0.61)	0.68 (0.16–2.95)	0.33 (0.16–0.68)	3.36 (0.46–24.40)	1.02 (0.58–1.76)
**V**	0.52 (0.11–2.39)	0.13 (0.04–0.40)	0.29 (0.16–0.51)	0.32 (0.19–0.52)	0.59 (0.13–2.65)	0.35 (0.16–0.76)	1.29 (0.17–9.91)	0.48 (0.25–0.91)
*No of injuries*	*63*	*94*	*249*	*316*	*62*	*135*	*98*	*168*
Self-inflicted								
**I**			1.00		1.00	1.00	1.00	1.00
**II**			1.04 (0.32–3.37)		1.38 (0.28–6.89)	1.41 (0.30–6.53)	0.70 (0.30–1.61)	3.67 (1.70–7.89)
**III**			1.24 (0.45–3.44)		2.24 (0.54–9.25)	1.70 (0.40–7.16)	1.01 (0.51–2.02)	2.77 (1.33–5.77)
**IV**			0.96 (0.34–2.66)		1.82 (0.44–7.52)	1.46 (0.35–6.13)	1.35 (0.69–2.64)	6.34 (3.11–12.92)
**V**			1.03 (0.37–2.90)		1.87 (0.45–7.77)	1.85 (0.43–7.93)	1.52 (0.77–2.98)	4.73 (2.30–7.93)
*No of injuries*	*48*	*16*	*142*	*59*	*127*	*82*	*384*	*343*
Violence-related								
**I**		1.00	1.00	1.00		1.00		1.00
**II**		1.24 (0.47–3.28)	0.77 (0.34–1.76)	0.85 (0.59–1.48)		1.57 (0.45–5.41)		1.96 (0.69–5.55)
**III**		1.44 (0.58–3.58)	0.72 (0.36–1.45)	1.01 (0.61–1.67)		2.12 (0.66–6.83)		3,36 (1.34–8.42)
**IV**		1.22 (0.49–3.03)	0.86 (0.43–1.70)	0.87 (0.53–1.44)		1.48 (0.46–4.77)		3,31 (1.32–8.33)
**V**		1.21 (0.47–3.10)	0.94 (0.47–1.88)	0.88 (0.52–1.47)		1.62 (0.49–5.36)		2,89 (1.13–7.40)
*No of injuries*	*28*	*181*	*253*	*446*	*12*	*131*	*35*	*148*

### Simple and partial correlations between indices and injury causes in two periods

Table 6 presents the simple and partial correlations between each index and cause of injury split into time period (1993–1995, 2003–2005), gender and age group. Quite a few causes of injuries are not significantly correlated with the indices when considering the simple correlations only.

**Table 6:** Correlation between indices and causes of injuries by period, gender and age group

	**Simple correlation**		**Partial correlation**	
	**Economic deprivation**	**Social fragmentation**	**Economic deprivation**	**Social fragmentation**
**First period (1993–1995)**				
***Girls 10–14 years***				
Fall	0.36**	0.36**	0.12	0.11
Vulnerable road user	0.16	0.15	0.06	0.04
Motor-vehicle rider	0.11	0.11	0.04	0.02
Self-inflicted	0.28**	0.32**	0.02	0.17*
Violence-related	0.12	0.13	0.03	0.05
***Girls 15–19 years***				
Fall	0.32**	0.34**	0.08	0.13
Vulnerable road user	0.20*	0.20*	0.06	0.06
Motor-vehicle rider	0.15	0.14	0.05	0.04
Self-inflicted	0.48**	0.47**	0.16*	0.16
Violence-related	0.28**	0.25**	0.12	0.05
***Boys 10–14 years***				
Fall	0.42**	0.40**	0.17*	0.10
Vulnerable road user	0.37**	0.37**	0.12	0.12
Motor-vehicle rider	0.01	0.07	-0.10	0.12
Self-inflicted	0.32**	0.34**	0.08	0.13
Violence-related	0.23**	0.22**	0.10	0.04
***Boys 15–19 years***				
Fall	0.37**	0.38**	0.12	0.13
Vulnerable road user	0.29**	0.31**	0.05	0.14
Motor-vehicle rider	0.15	0.13	0.08	0.01
Self-inflicted	0.29**	0.31**	0.05	0.14
Violence-related	0.37**	0.34**	0.18*	0.05
				
**Second period (2003–2005)**				
***Girls 10–14 years***				
Fall	0.41**	0.30**	0.30**	-0.12
Vulnerable road user	0.25**	0.17*	0.20*	-0.10
Motor-vehicle rider	0.11	0.06	0.12	-0.08
Self-inflicted	0.35**	0.28**	0.24**	-0.07
Violence-related	0.22**	0.33**	0.28**	-0.14
***Girls 15–19 years***				
Fall	0.30**	0.25**	0.16	-0.01
Vulnerable road user	0.26**	0.24**	0.11	0.03
Motor-vehicle rider	0.19*	0.09	0.22**	-0.14
Self-inflicted	0.53**	0.41**	0.39**	-0.11
Violence-related	0.30**	0.23**	0.22**	-0.07
***Boys 10–14 years***				
Fall	0.37**	0.27**	0.27**	-0.09
Vulnerable road user	0.28**	0.19*	0.23**	-0.10
Motor-vehicle rider	0.13	0.06	0.17*	-0.12
Self-inflicted	0.30**	0.22**	0.23**	-0.08
Violence-related	0.21*	0.32**	0.28**	-0.14
***Boys 15–19 years***				
Fall	0.49**	0.35**	0.38**	-0.14
Vulnerable road user	0.21*	0.18*	0.10	0.01
Motor-vehicle rider	0.22**	0.13	0.21*	-0.12
Self-inflicted	0.44**	0.34**	0.30**	-0.08
Violence-related	0.42**	0.30**	0.33**	-0.13

* p < 0.05; ** p < 0.01.

The compilation of partial correlations reveals remarkable changes both within index and between periods. During the first period, very few correlations remain significant, but three exceptions are noteworthy as they concern intentional injuries: the association between economic deprivation and self-inflicted injuries among older girls, that between economic deprivation and violence-related injuries among older boys and that between social fragmentation and self-inflicted injuries among younger girls. During the second period, one can see that, once correction for economic deprivation has been made none of the injury causes correlated significantly with the index of social fragmentation. When correction had been made for social fragmentation, nearly all causes correlated significantly with economic deprivation. The exceptions concern unintentional injuries as vulnerable road users, among older boys and girls, injuries as motor-vehicle riders among young girls and falls among older girls.

## Discussion

This study is one of the very few investigating variability in the social patterning of childhood injuries over time [31]. It considers two area-based aspects offering different but complementary contextual perspectives of the social fabric of a parish/living area and considers their individual and independent association with various injury causes. Another distinctive feature is that the study places focus on children in the second decade of life rather than on pre-school children or those aged 0–15 years. This is partly because, in Sweden, injury rates tend to increase considerably during that time [11,15].

### Main findings

Over the time period covered, the range of material deprivation varied more than the range of social fragmentation. Also, whereas about half of the parishes remained at the same level for the two indices, both improvements and deteriorations occurred; improvements tended to be more frequent in the northern part of Stockholm County. An additional noteworthy change is that violence-related injuries and injuries as motor-vehicle riders were substantially higher in 2004 than they were in 1994.

One striking finding of is that, in both time periods, when each index is considered separately, there is no clear association between either material deprivation or social fragmentation and injuries. This is in stark contrast to findings from studies on pediatric injuries conducted in other high-income countries, for instance in the UK [26], the US [31] or New Zealand [27] where substantial differences are observed between children from deprived areas compared to those from better-off ones.

In addition, when associations are found, mixed results are observed and both protective and aggravating effects were noted; they also tended to be gender- and age-specific. For instance, in each period, boys from both age groups living in parishes with the highest levels of economic deprivation had lower rates of injury as motor vehicle riders than boys from better-off parishes (as if deprivation would act as a protective factor). Conversely – and most strikingly – intentional injuries were not only more frequent during the second time period but also in considerable excess among girls aged 15–19 from more economically deprived areas (as if deprivation would act as an aggravating factor).

Earlier studies from Stockholm County conducted on children aged 0–15 revealed substantial parish-based differences in injury risks of various kinds with regards to contextual measures describing population socioeconomic position and material deprivation [34,42]. The exposures chosen were additive indices based on a number of selected rather than pre-determined indices. Similar to this study, those earlier studies revealed a number of associations but no consistent patterns. For instance, higher levels of material deprivation were negatively associated with pedestrian injury, but positively with other traffic-related injuries. Higher concentrations of people with low socioeconomic status did not impact on the risk of fall and traffic injuries, but increased the risk of burns/scalds and poisoning.

Also in line with our findings among 16–19 year-old girls, a study from Wales which used the Townsend index of deprivation, found that socioeconomic variations in injury rates were much smaller in older people (15+ years) than in children (0–14 years). The largest variations were for injuries sustained in assaults or self-inflicted ones [29]. A possible explanation for this relatively smaller effect of deprivation on older children/youth is that factors other than material deprivation in the living area play a more determinant role on their health and wellbeing, including factors relating to youth culture, peer group and school contexts [43].

For social fragmentation, the patterns observed are similar in many ways to those found for economic deprivation. This is definitely the case for the two categories of intentional injuries and for traffic-related injuries as vulnerable road users (all instances) and motor-vehicle riders among boys.

Finally, the compilation of partial correlations has helped us to disentangle the relative importance of each index. Those correlations reveal substantial changes both within index and between periods. In particular, during the second period, none of the injury causes correlated significantly with the index of social fragmentation after correcting for the level of economic deprivation. Moreover, once correction had been made for social fragmentation, nearly all causes correlated significantly with economic deprivation. The exceptions concern unintentional injuries as vulnerable road users, among older boys and girls, injuries as motor-vehicle riders among young girls and falls among older girls.

## Limitations

As the study is ecological, it is not possible to determine whether the results could apply to individual children aged 10–19 – or to their family units. Studies conducted on health outcomes like cardiac disease [50], childhood injuries [51] and poverty [52] suggest however that whereas area and individual material deprivation are not interchangeable measures, both capture important health determinants.

Also, as the observation unit – a parish – is rather large, one could question whether it is a fair representation of a neighborhood. This may have contributed to non-differential misclassification, biasing our rate ratios towards unity. The relevance of the parish as an observation unit varies depending on the outcome chosen. In studies conducted in the Stockholm metropolitan area at parish level, there are indications that variability across parishes is sufficiently high to investigate health outcomes like myocardial infarction [36] and several – but not all (e.g., falls) – injury causes [33,34].

Further, the analyses were conducted under the assumption that residential mobility is likely to be in similar proportions across parishes. If this is not the case, there might be a misclassification bias that would dilute the main associations.

Further, the fact that the index of material deprivation includes the percentage of car ownership in the parish, conclusions ought to be drawn cautiously. Used as a measure of material deprivation, car ownership becomes a measure of both material deprivation in the parish as well as of potential exposure of children in the traffic environment. Because of this, as car ownership increases, it is possible that injuries as car riders tend to increase and those as vulnerable road users tend to decrease. In the current study, boys in the 10–19 age group in both periods and 10–14 year-old girls in the 93–94 period demonstrated an under-risk in motor vehicle-related accidents in the most deprived areas. Almost exactly the same results were shown in the study (in which factor analysis was used) on moped accidents in areas with an average and high socioeconomic precariousness and ethnic concentration factor (including car ownership) [33]. But it ought to be underlined that car ownership in the parish is by no means a summary of potential exposure in the area: it does not mean that the children injured as vulnerable road users are those owning cars (or their parents), it does not inform about the road traffic infrastructure in the area, and it does not tell either how well the roads in an area are used by its inhabitants and by other road users (e.g., central Stockholm).

Our study also suffers from a lack of control for the confounding effects of exposures related to the physical characteristics of living environments that may reduce the rate ratios. Other types of information of documented relevance are the health-care environment, community standard of living, and economic vitality [40].

The extent to which the results would apply to the same extent to injury mortality – or for that matter injuries not leading to hospitalization – is uncertain. The data at hand cover hospitalization cases only, of which a relatively small proportion is expected to be fatal. Data on fatal injuries are included in the Swedish death register, to which we did not have access in this study. It is estimated that, yearly in Stockholm County for 200 injured inpatients aged 0–17, there will be about 4 000 outpatients and one death.

In relation to time, quite a few changes have happened in the county between the two reference periods. As presented earlier, some have to do with the exposures (range and distribution of the parishes) and some with outcome (increases in some diagnoses and reductions in other). In the latter case, we have no possibility to determine whether differences in exposures only or differences in practice in the hospitals and referral system are responsible for what is observed. Even the manner in which injuries are classified has moved from the 9^th ^to the 10^th ^revision of the International Classification of Diseases. During this period more patients have received their care in a polyclinic instead of being inpatients. This certainly affects statistics of all parishes in our study. But we are sure that it happens quite equally. Besides, we are not interested in absolute terms but relative differences between groups of parishes over time. As we are more interested in relative rather than absolute differences and as we have no reason to believe these changes would differentially affect children from different areas, we trust that our results are not strongly affected by those changes.

Differences in access to medical care or in healthcare consumption on the part of the child (and family), combined with misclassification on the part of hospital staff – in particular, possible systematic bias in the case of violence-related injuries – may mean that the actual rate ratios are larger than they have been presented [9,15].

### Implications for policy and research

Our result suggest that area-based material deprivation and social fragmentation are not strongly associated with the injury causes considered, suggesting that injury prevention may not need in all instances to be tailored in some particular way at area level. This does not imply however that children from families living in less favorable economic and social conditions do not need particular attention (ecological fallacy). Also, some injury causes may need both family and area-based investments, in particular injuries as motor-vehicle riders among boys aged 15–19 years and self-inflicted injuries among girls aged 15–19 years. The increasing numbers of violence-related injuries deserve particular attention.

The mechanism lying behind the associations observed deserves special attention.

## Conclusion

In this study, we have disentangled the effects of neighborhood material deprivation and social fragmentation on injuries among boys and girls during two periods. Since 1994, in the Stockholm County area, differential economic deprivation among parishes has widened more than social fragmentation. Among children aged 10–19 years, there is no clear social patterning of injuries from various causes considering both indices. Yet, intentional injuries have increased and they are significantly and positively correlated with the economic deprivation net of social fragmentation, in particular among girls.

## Competing interests

The authors declare that they have no competing interests.

## Authors' contributions

AMR participated in the design and coordination of the study, carried out all statistical analyses, and drafted the manuscript. APL participated in the design, supervision of the study, as well as providing statistical guidance. LL participated in the design and coordination, and supervision of the study, as well as revision of the manuscript. All authors read and approved the final manuscript.

## Pre-publication history

The pre-publication history for this paper can be accessed here:


